# An Evaluation of Comparability between NEISS and ICD-9-CM Injury Coding

**DOI:** 10.1371/journal.pone.0092052

**Published:** 2014-03-21

**Authors:** Meghan C. Thompson, Krista K. Wheeler, Junxin Shi, Gary A. Smith, Huiyun Xiang

**Affiliations:** 1 Center for Injury Research and Policy, The Research Institute at Nationwide Children’s Hospital, Columbus, Ohio, United States of America; 2 The Ohio State University College of Medicine, Columbus, Ohio, United States of America; University of Western Australia, Australia

## Abstract

**Objective:**

To evaluate the National Electronic Injury Surveillance System’s (NEISS) comparability with a data source that uses ICD-9-CM coding.

**Methods:**

A sample of NEISS cases from a children’s hospital in 2008 was selected, and cases were linked with their original medical record. Medical records were reviewed and an ICD-9-CM code was assigned to each case. Cases in the NEISS sample that were non-injuries by ICD-9-CM standards were identified. A bridging matrix between the NEISS and ICD-9-CM injury coding systems, by type of injury classification, was proposed and evaluated.

**Results:**

Of the 2,890 cases reviewed, 13.32% (n = 385) were non-injuries according to the ICD-9-CM diagnosis. Using the proposed matrix, the comparability of the NEISS with ICD-9-CM coding was favorable among injury cases (κ = 0.87, 95% CI: 0.85–0.88). The distribution of injury types among the entire sample was similar for the two systems, with percentage differences ≥1% for only open wounds or amputation, poisoning, and other or unspecified injury types.

**Conclusions:**

There is potential for conducting comparable injury research using NEISS and ICD-9-CM data. Due to the inclusion of some non-injuries in the NEISS and some differences in type of injury definitions between NEISS and ICD-9-CM coding, best practice for studies using NEISS data obtained from the CPSC should include manual review of case narratives. Use of the standardized injury and injury type definitions presented in this study will facilitate more accurate comparisons in injury research.

## Introduction

Injury is a leading cause of death and disability in children and surveillance is an essential component of injury prevention [1]–[4]. The National Electronic Injury Surveillance System (NEISS) has served as a data source for many studies and provides nonfatal injury data for the Centers for Disease Control’s Web-based Injury Statistics Query and Reporting System [5]–[9]. The NEISS monitors injuries treated in US hospital emergency departments (EDs) [10]. While previous authors have identified limitations and evaluated sub-categories of the NEISS design, [6], [7], [11]–[15] no study has comprehensively evaluated the NEISS injury definition or diagnosis coding system. Furthermore, because the NEISS uses its own unique coding system and inclusion criteria, it is unknown whether injury statistics from NEISS are readily comparable to results based on the *International Classification of Diseases, Ninth Revision, Clinical Modification* (ICD-9-CM) codes.

Identifying specific differences in diagnosis classification between the NEISS and an ICD-9-CM standard has implications for the interpretation of injury literature. Standardized coding and presentation of data, injury definitions, and injury type classifications facilitate comparability and offer cost-savings in the areas of comparing, linking and analyzing data [1]. In the US, hospital records are coded using the ICD-9-CM guidelines [1]. The Barell matrix, a current standard for injury data collection, analysis and presentation, also uses ICD-9-CM codes to organize injuries by injury type and body part injured [1], [16], [17]. _ENREF_3 Given its prominent role in data coding and research standards, ICD-9-CM coding may be used as a benchmark against which other injury coding systems are evaluated. A study of traumatic brain injury (TBI) definitions used in NEISS studies assigned ICD-9-CM codes to 1,018 NEISS cases of all diagnoses and found that only 880 were injuries according to the ICD-9-CM code [11]. However, this earlier study focused on TBI case identification and did not explore other NEISS injury definitions [11].

A bridging matrix between the existing NEISS and ICD-9-CM injury classification systems would provide a framework for conducting comparable injury research using the NEISS, without incurring the high costs associated with overhauling the already well-established NEISS system [6]. A few previous publications “map” ICD-9-CM diagnosis codes to corresponding NEISS body part and diagnosis codes, with the aim of using data from different sources to estimate injury costs [18]-[20] or conduct uncertainty analyses [21]. A 2011 report briefly explains and uses a map between ICD-9-CM diagnosis and NEISS diagnosis and body part codes that was developed during a previous cost-estimation study; [18], [19] however, the map is neither described in-depth nor published in the previous study [19]. A 2010 report also uses a “pre-existing map” between NEISS and ICD-9-CM codes from a 1995 publication [20], [21]. However, while Miller et al. (1995) detail the development, importance, and use of “injury code maps” or “equivalency tables” between ICD and NEISS codes, along with some maps between ICD-9 (used as a sort of standard for coding) and other coding systems, the actual tables are published separately and are not readily accessible [20].

The main objective of this study is to propose and evaluate a bridging matrix, by type of injury classification, between the NEISS and ICD-9-CM coding systems. By linking pediatric records from the NEISS with their original medical records, this study investigates differences in the injury and injury type definitions and aims to provide a general, accessible framework for researchers. Fulfillment of these objectives will elucidate some limitations and applications of a frequently used data source and advance the development of injury definitions and classifications that are compatible across multiple data sources.

## Methods

### Ethics statement

Because we linked our site’s NEISS cases to data from the hospital’s electronic medical record (EMR), Institutional Review Board (IRB) approval was obtained through a full human subject protection review by the Nationwide Children’s Hospital IRB. Written consent was not obtained, and it was waived by the approving IRB.

### Data sources

The NEISS database is maintained by the Consumer Product Safety Commission (CPSC) and collects data from a stratified, national probability sample of approximately 100 US emergency departments (EDs) [10]. NEISS coders review electronic medical records (EMRs) from the ED and enter visits meeting the inclusion criteria into the NEISS database [22]. The NEISS has its own unique coding system, and injuries are assigned both a diagnosis code and body part code [22]. The NEISS has monitored product-related injuries since 1971, but was expanded in 2000 to monitor all injuries, not just those related to specific consumer products [10], [23]._ENREF_22 The NEISS All Injury Program (NEISS-AIP) uses a subsample of the NEISS participating emergency departments for its data collection. We evaluated data collected at Nationwide Children’s Hospital, one of 66 NEISS hospital EDs that participates in the NEISS-AIP [23]. As a consequence of its role as a NEISS-AIP site, this study includes data on all injuries, not just consumer product-related injuries.

The NEISS-AIP is a collaborative effort between the CPSC and the National Center for Injury Prevention and Control, the Centers for Disease Control (CDC) [23]. The CDC excludes non-injury cases in its NEISS-AIP public release data files and Web-based Injury Statistics Query and Reporting System (WISQARS). The site-specific NEISS data used in this study was obtained directly from the CPSC and contained additional data not available in the NEISS-AIP public use data files and WISQARS estimates because CDC excludes non-injury cases in their public release of the data (personal communication, July 2013) [24]. For this reason, the research team did its own review to identify non-injury cases. Specifically, the NEISS-AIP public use data files exclude cases which are included in our NEISS dataset obtained from the CPSC including “illness,” “psychological harm only,” “contact dermatitis…associated with exposure to consumer products… or plants,” “pain symptoms…[without an injury-related diagnosis specified],” “visit [for] adverse effects of therapeutic drugs or of surgical and medical care,” unknown diagnoses, and deaths occurring upon arrival or in the ED [23].

A random sample of 10% of each month’s NEISS cases seen at the study hospital between January 1, 2008 and December 31, 2008 was selected (n = 3,000). These NEISS records were then linked with their corresponding EMR using the date of treatment (+/– 1 day) and the patient medical record number (matched n = 2,992). Because the treatment date was loosened to +/– 1 day during the match, some EMR entries were inappropriately matched to a NEISS record if a patient visited the ED on two consecutive days. These inappropriately matched cases were removed after manually reviewing the EMR. Additionally, off-site urgent care records (which are not entered in the NEISS but were in the EMR data) and follow-up visits were eliminated. Children were defined as ≤18 years old; consequently, 48 records were eliminated because the patients were ≥19 years. The final database contained 2,890 matched cases.

### Diagnosis code review and the definition of injury

Drawing upon the review methodology used in a previous NEISS and ICD-9-CM comparison TBI study, [11] the EMR for each case was reviewed separately by two research team members trained in ICD-9-CM coding. Each researcher assigned one final ICD-9-CM diagnosis code for an individual case. Multiple injuries, burns, and poisonings required additional consideration to select the most serious injury and/or appropriate final ICD-9-CM code.

The benchmark ICD-9-CM standard definition of injury used in this study drew upon the

ICD-9-CM injury codes used in the Barell matrix, a current standard for injury data collection, analysis and presentation [16], [17]. Following the Barell matrix, this study considered all diagnoses within the 800-999 range as injuries, with the exception of 909(.3,.5): “Late effect of complications of surgical and medical care, late effect of adverse effect of drug, medicinal or biological substance”; 995(.0-.4,.6,.7,.86,.89): “Certain adverse effects, not elsewhere classified”; 995.9 “Systemic inflammatory response syndrome” and 996-999: “Complications of surgical and medical care, not elsewhere classified” [25]. Cases not assigned a final code falling within this injury definition were considered non-injuries. Table 1, column 3 presents the ICD-9-CM codes included within the benchmark injury definition used in this study.

**Table 1:** Bridging matrix between the NEISS and ICD-9-CM coding systems, by type of injury.

Type of injury	NEISS diagnosis code	Corresponding ICD-9-CM injury code*
**Burns**	46–49, 51, 73	940–949
**Traumatic brain injury**	52, 62+B75**, 57+B75	800, 801, 803, 804, 850.0–854, 950(.1-.3), 995.55, 959.01
**Soft tissue injury**	53, 58	910(.0,.1), 911(.0,.1), 912(.0,.1), 913(.0,.1), 914(.0,.1), 915(.0,.1), 916(.0,.1), 917(.0,.1), 918(.0,.1,.9), 919(.0,.1), 920–924
**Foreign body**	41, 42, 56	910 (.6,.7), 911 (.6,.7), 912 (.6,.7), 913 (.6,.7), 914 (.6,.7), 915 (.6,.7), 916 (.6,.7), 917 (.6,.7), 919 (.6,.7), 930–939
**Dislocation**	55	830–839
**Fracture**	57 (except 57+B75)	802, 805–829
**Open wound or amputation**	50, 59, 60, 63, 72	870–897
**Internal organ injury**	62 (except 62+B75)	860–869, 952
**Poisoning**	68	960–989
**Sprain or strain**	64	840–848
**Blood vessels or nerve**	66, 61	900–904, 950.0, 950.4–951, 953–957
**Crush**	54	925–929
**Other or unspecified**	65, 67, 71, 74, 69	905–908, 909 (.0,.1,.2,.4,.9), 910(.2,.3,.4,.5,.8,.9), 911(.2,.3,.4,.5,.8,.9), 912(.2,.3,.4,.5,.8,.9), 913(.2,.3,.4,.5,.8,.9), 914(.2,.3,.4,.5,.8,.9), 915(.2,.3,.4,.5,.8,.9), 916(.2,.3,.4,.5,.8,.9), 917(.2,.3,.4,.5,.8,.9), 918.2, 919(.2,.3,.4,.5,.8,.9), 958, 959.0–959.9 (excluding 959.01), 990–994, 995.50–.54, 995.59, 995.80–995.85

*A corresponding ICD-9-CM code is provided only if the ICD-9-CM code falls within the injury definition according to the ICD-9-CM standard definition developed by the research team. Therefore, the ICD-9-CM codes provided exclude non-injuries. For example, although dermatitis is included in the NEISS (diagnosis code 74), it is not an injury according to ICD-9-CM standards, and the ICD-9-CM code for dermatitis is not included in this matrix.

**B  =  NEISS “body part” code.

If the case was determined to be a “non-injury” by ICD-9-CM standards, no code was assigned. The codes assigned by the two research team members were compared, and codes that did not agree within three digits were re-reviewed together by the research team before assigning a final ICD-9-CM code or classifying the case as a non-injury. An ICD-9-CM coding manual and software were used [25], [26].

#### Bridging the NEISS and ICD-9-CM diagnosis codes

The NEISS diagnosis codes were grouped into thirteen injury type categories (Table S1) [22]. The categories were based upon nature of injury type categories in the Barell matrix, [16], [17] with some adjustments, mostly based upon how the NEISS codes certain cases [22]. The NEISS definition for TBI used was recommended in a previous study [11]. Only ICD-9-CM codes which met this study’s ICD-9-CM injury definition were placed into a matrix cell. For example, the NEISS burn definition includes sunburns, but the corresponding ICD-9-CM code for sunburn is outside the injury code range and is therefore not included in the matrix.

### Data analysis

Statistical analyses were conducted using SAS 9.3 (SAS Institute, Cary, NC). The percentage of non-injury cases in the NEISS sample was calculated by comparing the final ICD-9-CM code assigned by the research team against this study’s ICD-9-CM benchmark injury definition. Additionally, the percentage agreement and kappa coefficient for agreement were calculated to compare the injury versus non-injury classification of each case according to the research team-assigned ICD-9-CM code and the hospital-assigned ICD-9-CM code. Codes with the same first three digits “agreed.”

The proposed matrix was evaluated by calculating a cross-tabulation of the injury type classification under the ICD-9-CM system by the injury type classification under the NEISS (non-injuries excluded). Based on this cross-tabulation, a kappa coefficient was calculated. Using the entire sample (injury and non-injury cases), percentage differences between the injury type distributions of the two systems were calculated as the NEISS proportion minus the ICD-9-CM proportion.

## Results

### Injury Identification by the NEISS, Hospital and ICD-9-CM Diagnosis Coding

Of the 3,000 NEISS cases originally selected for this study, 2,890 cases were non-duplicate pediatric cases that were able to be linked to their original medical record. Of the 2,890 cases reviewed, 2,505 (86.68%) were injuries based upon the research team-assigned ICD-9-CM code. The remaining 385 cases (13.32%) were determined to be non-injuries because they did not meet this study’s ICD-9-CM benchmark injury definition. An additional analysis of all of the cases classified as non-injuries by the ICD-9-CM standard revealed that 93.5% (n = 360 out of 385) of all non-injuries were coded with at least one NEISS product code. Sports or recreation-related codes (n = 72), diapers (n = 66), medical equipment (general, n = 61), liquid drugs (excluding aspirin, aspirin substitutes, iron preparations and antihistamines, n = 46), motor vehicles or parts (licensed, four or more wheels, n = 22), and other drugs or medications (n = 20) were the products most frequently related to ICD-9-CM non-injuries. (For specific codes used, see Table S2.)

To gain an understanding of case ascertainment under different coding systems, we examined the sensitivity of injury identification using three different diagnosis codes: the NEISS diagnosis code, the research team-assigned ICD-9-CM codes, and the hospital-assigned ICD-9-CM codes. Because the sample was gathered from the NEISS, all 2,890 cases were injuries according to the NEISS. The research team identified the next greatest number of injuries (n = 2,505), and the hospital coders identified the least number of injuries (n = 2,121). Of 2,890 NEISS cases, 769 (26.6%) did not have a hospital-assigned ICD-9-CM code meeting the benchmark injury definition.

Given the variations in injury identification, the relationship between the research team-assigned and hospital-assigned ICD-9-CM codes was further explored. All 2,121 cases that were injuries according to the hospital code were also identified as injuries by the research team; however, the research team found an additional 384 injuries. The kappa coefficient between the hospital injury classification (i.e., injury or non-injury) and the research team classification was 0.60 (95% CI: 0.56-0.63). More specifically, the percentage agreement between the research team-assigned ICD-9-CM code and any of the ICD-9-CM codes assigned by the hospital coders was 77.41% (1,939 of 2,505 cases).

### Comparability of the NEISS and the ICD-9-CM classification systems

The main objective of this study was to develop and evaluate a bridging matrix between the ICD-9-CM and NEISS coding systems. Using the matrix and injury type classifications described in the methods (see Table 1), we calculated a cross-tabulation of the injury type according to the ICD-9-CM classification by the injury type according to the NEISS classification. Only injury cases were used in this analysis (n = 2,505) (Table 2). Among the injury cases, overall agreement for injury type between the two classification systems was high (κ = 0.87, 95% CI: 0.85–0.88), although some discrepancies existed among certain injury type categories. Overall totals for soft tissue injuries were similar for both the ICD-9-CM (n = 292) and NEISS systems (n = 295), but only 231 cases were soft tissue injuries under both (Table 2). Many injuries coded as soft tissue injuries by the NEISS were classified as other or unspecified injuries under the ICD-9-CM system (n = 42), while some cases coded as soft tissue injuries under ICD-9-CM were also coded as other or unspecified (n = 26) injuries under the NEISS. Similarly, totals for other or unspecified injuries were similar under the ICD-9-CM (n = 450) and NEISS systems (n = 456), but only 360 overlapped. The ICD-9-CM code classifies many NEISS “other” injuries as TBI (n = 18), soft tissue (n = 26), and open wound or amputation (n = 28) injuries. The NEISS classifies many ICD-9-CM “other” injuries as soft tissue (n = 42) and sprain or strain (n = 20) injuries (Table 2).

**Table 2:** Comparability of NEISS and ICD-9-CM Code classification systems.

Type of Injury: ICD-9-CM Code Classification	Type of Injury: NEISS Code Classification														
	Burns	TBI	Soft tissue injury	Foreign body	Dislocation	Fracture	Open wound or amputation	Internal organ injury	Poisoning	Sprain or strain	Blood vessels or nerve	Crush	Other or unspecified	Total	Cases that match as a % of total cases identified by ICD-9-CM
Burns	46		2										1	49	93.9
TBI		228	5			1	3	1					18	256	89.1
Soft tissue injury		12	231	2		2	13		1	3	2		26	292	79.1
Foreign body	1		1	98			1	1					4	106	92.5
Dislocation					54	1								55	98.2
Fracture			1			335	4			1			7	348	96.3
Open wound or amputation		6	9	6		2	633	2	2	1	1		28	690	91.7
Internal organ injury			1					6						7	85.7
Poisoning			1						76				7	84	90.5
Sprain or strain			1			2				157			5	165	95.2
Blood vessels or nerve											1			1	100.0
Crush			1									1		2	50.0
Other or unspecified	2	1	42	1	4	5	2	5		20	8		360	450	80.0
Total	49	247	295	107	58	348	656	15	79	182	12	1	456	2505*	
Cases that match as a % of total cases identified by NEISS	93.9	92.3	78.3	91.6	93.1	96.3	96.5	40.0	96.2	86.2	8.3	100.0	78.9		

*Sample includes only cases classified as injuries according to the ICD-9-CM definition determined by the research team. The table and kappa statistic do not include the 385 NEISS cases classified as non-injuries.

Kappa Coefficient for Agreement  =  0.87 (95% CI: 0.85–0.88).

Because the cross-tabulation and agreement analysis was limited to injury cases, the percentage differences between the type of injury distributions of the two classification systems were calculated to provide a comparability measure for the total sample (injury and non-injury cases, n = 2,890) (Table 3). Percentage differences (the NEISS minus the ICD-9-CM percentages) were minimal for most injury types, with the exception of open wound or amputation (–1.08%), poisoning (2.63%), and other or unspecified (9.27%) cases. Under the NEISS categories of poisoning and other or unspecified, there were significant numbers of ICD-9-CM non-injuries (e.g. adverse reactions to pharmaceuticals and medical devices, dermatitis, and conjunctivitis). The matrix analysis presented in Table 2 shows that, among injury cases, open wound or amputation injuries are coded more frequently under the ICD-9-CM system, and that the NEISS categorizes many of these injuries as other or unspecified (Table 2, n = 28).

**Table 3:** Percentage distribution of type of injury, by classification system.

Type of Injury	ICD-9-CM Classification of injury N = 2890		NEISS Classification of injury N = 2890		Difference in the Percentages
	n	%	n	%	
**Burn**	49	1.70	55	1.90	0.20
**TBI**	256	8.86	249	8.62	–0.24
**Soft tissue injury**	292	10.10	299	10.35	0.25
**Foreign body**	106	3.67	120	4.15	0.48
**Dislocation**	55	1.90	58	2.01	0.11
**Fracture**	348	12.04	349	12.08	0.04
**Open wound or amputation**	690	23.88	659	22.80	–1.08
**Internal organ injury**	7	0.28*	18	0. 62*	*
**Poisoning**	84	2.91	160	5.54	2.63
**Sprain or strain**	165	5.71	186	6.44	0.73
**Blood vessel or nerve**	1	0.04*	18	0.62*	*
**Crush**	2	0.08*	1	0.03*	*
**Other or unspecified**	450	15.57	718	24.84	9.27
**Non-injury case** **	385	13.32	0	0.00	–13.32

*Sample size is too small to calculate accurate percentages.

**Non-injury case according to the ICD-9-CM code benchmark definition of injury determined by the research team.

## Discussion

The NEISS is a frequently used database that plays a critical role in national-level injury research and surveillance [10], [22]. Although the need to evaluate injury surveillance systems and to have standardized definitions across data sources have been recognized, [1],[27],[28] no study has comprehensively evaluated the NEISS’s diagnosis coding system. To our knowledge, this is the first study to go beyond reports of limitations and evaluations of selected NEISS sub-categories [6], [7], [11]–[15] and comprehensively evaluate the general comparability potential of NEISS data with data sources that use ICD-9-CM diagnosis coding. Although previous studies have successfully mapped between NEISS and ICD-9-CM diagnoses [18]–[21] and a code equivalency table between NEISS and ICD-9-CM exists, [20] this study improves upon previous work by providing an accessible and easy-to-understand framework for interpreting and conducting injury research using NEISS and ICD-9-CM codes. This study’s most important findings, such as the NEISS’s promising comparability potential with ICD-9-CM coding and the frequency of certain NEISS injury types and product codes among non-injuries, have important implications for the standardized interpretation and conduct of injury research.

The bridging matrix presented in this study provides a basic framework for conducting injury surveillance research. Using data from injury cases only, agreement between the proposed NEISS and ICD-9-CM classification systems was promisingly high (k = 0.87). Of particular note, TBI injuries demonstrated a good level of agreement: 247 cases were identified by the NEISS and 256 cases were identified with the ICD-9-CM research team code, with 228 overlapping cases. This result supports previous findings that the NEISS is a relatively good source for TBI data [11]. As described in the results, small comparability issues arose in the soft tissue injury and other or unspecified injury type categories.

Using the classifications presented in the matrix, comparability among the entire NEISS sample (injuries and non-injuries), measured in terms of percentage differences between injury type proportions, was also favorable. Only open wound or amputation, poisoning, or other or unspecified injuries had percentage differences >1%, and the largest percentage differences (poisonings, 3% and other or unspecified injuries, 9%) were consistent with the injury type profile observed for non-injuries. In a sub-analysis of the 385 non-injuries in our sample, we found that according to the NEISS diagnosis code, a majority (nearly 70 percent) of ICD-9-CM non-injuries fell within the “other or unspecified” injury type, and approximately one-fifth of the non-injuries were NEISS poisoning cases.

Because of NEISS’s primary focus on consumer product-related injuries [10] it includes both injuries and adverse events that are non-injuries. An ED visit is captured by the NEISS even if it the diagnosis is not technically an injury, such as an “illness,” “disorder,” or observation, but a consumer product is noted to be “associated” with the “onset” [29]. Investigation of the non-injuries in our sample revealed that NEISS studies, especially those focusing on diapers, sports or recreation-related codes, liquid drugs, motor vehicles or parts, and other drugs or medications, should be aware of the possibility of these non-injuries. CPSC, in order to not overly complicate the reporting rules, collects through NEISS more cases than are ultimately released (personal communication, September 2013) [24]. Still, users of the NEISS data obtained from the CPSC may need to manually review the data when the exclusion of these non-injuries is desired. Users of the NEISS-AIP data (prepared by the CDC) and estimates available in WISQARS will be working with data from which the non-injuries have been removed [23].

Additionally, there were large variations in injury ascertainment when comparing injury identification using the research-team assigned ICD-9-CM codes and the hospital-assigned ICD-9-CM codes. Nearly 27 percent of the NEISS sample cases were non-injuries according to the hospital-assigned codes, compared to thirteen percent based on the research team’s ICD-9-CM codes. From a pragmatic perspective, this result has implications for researchers who cull an injury sample based upon hospital-assigned ICD-9-CM codes. Our results suggest that using only hospital-assigned ICD-9-CM codes may exclude some injury cases and introduce a negative bias. Although the reason for the much lower injury ascertainment by hospital-assigned codes is not certain, NEISS coders may err on the side of over-inclusion so as not to miss potential injuries, while hospital coders may identify fewer injuries because they are not specifically looking for injuries and because they are generating codes primarily for billing rather than research purposes.

### Limitations

Our study has some limitations. Like all chart reviews, [6] assignment of an accurate ICD-9-CM code depended upon details available in the EMR. Because code assignment from medical records is sometimes subjective, [30] this study attempted to mitigate subjectivity by having two researchers review each case separately and assign a diagnosis code. A certain level of inter-rater agreement (a recommended practice for chart reviews) was required, [30] and questionable cases were discussed as a team. Also, using the research team’s ICD-9-CM codes instead of the hospital-assigned ICD-9-CM codes provided an additional quality control. Additionally, although ICD-9-CM codes are still used to code billing data in the US, the US is scheduled to switch to ICD-10-CM coding in October 2014 [31]. _ENREF_20

Although quality reviewers at the CPSC code cause of injury for NEISS data in a fashion that is “consistent” with ICD-9-CM external cause of injury codes (E-codes), [32] this study only examined comparability based upon non-supplementary ICD-9-CM diagnosis codes and NEISS diagnosis codes. Also, while previously used maps provided corresponding combinations of both NEISS diagnosis and body part codes to ICD-9-CM codes, [18]–[21] this study used only the NEISS diagnosis code (except for traumatic brain injuries, which used only the body part equal to the head).

Data limitations may affect the generalizability of results. The data used in this study comes from a single institution. If variations in coding exist across hospitals, then the external validity of these results is limited; however, no current indications suggest that the NEISS coding or design issues identified in this study are particular to our hospital. Additionally, it is unknown if these results are generalizable to adults or non-pediatric EDs.

### Conclusion

By proposing and evaluating a relatively simple matrix that bridges NEISS diagnosis codes with ICD-9-CM codes, this study provides a basic framework for conducting standardized injury research. Although comparability was imperfect, the generally favorable matrix evaluation results suggest that the NEISS has good comparability potential. Strategies such as manually reviewing the selected NEISS cases may further improve comparability. Additionally, the identified differences in injury and injury type definitions between NEISS and ICD-9-CM coded data will allow researchers to more accurately interpret NEISS results and pay attention to specific criteria of the NEISS when conducting research.

## Supporting Information

Table S1 **NEISS diagnosis code descriptions, by type of injury category.**
(DOCX)

Table S2 **Most common NEISS product codes among ICD-9-CM non-injuries.**
(DOCX)
