# Supplementary material for: An Evaluation of Comparability between NEISS and ICD-9-CM Injury Coding
Source: PLoS One. 2014 Mar 21;9(3):e92052. doi: 10.1371/journal.pone.0092052 (PMC3962381; doi:10.1371/journal.pone.0092052)
Supplement: Table S1 — NEISS diagnosis code descriptions, by type of injury category. (DOCX) [file pone.0092052.s001.docx]

**Table S1. NEISS diagnosis code descriptions, by type of injury category**

| Type of injury category | NEISS diagnosis code and code description [22] |
| --- | --- |
| Burns | 46: burns, electrical; 47: burns, not specified; 48: burns, scald (from hot liquids or steam); 49: burns, chemical (caustics, etc.); 51: burns, thermal (from flames or hot surface); 73: burns, radiation (includes all cell damage by ultraviolet, x-rays, microwaves, laser beam, radioactive materials, etc.) |
| Traumatic brain injury | 52: concussions; 62 + B75*: internal organ injuries to the head; 57 + B75: fractures to the head |
| Soft tissue injury | 53: contusions, abrasions; 58: hematoma |
| Foreign body | 41: ingested foreign object; 42: aspirated foreign body; 56: foreign body |
| Dislocation | 55: dislocation |
| Fracture | 57 (except 57 + B75): fractures, except fractures to the head |
| Open wound or amputation | 50: amputation; 59: laceration; 60: dental injury; 63: puncture; 72: avulsion |
| Internal organ injury | 62 (except 62 + B75): internal organ injury, except internal organ injuries to the head |
| Poisoning | 68: poisoning |
| Sprain or strain | 64: strain or sprain |
| Blood vessels or nerve | 66: hemorrhage; 61: nerve damage |
| Crush | 54: crushing |
| Other or unspecified | 65: anoxia; 67: electric shock; 71: other/not stated; 74: dermatitis, conjunctivitis; 69: submersion (including drowning) |

*B75 = NEISS “body part” code for head
