# Supplementary material for: An Evaluation of Comparability between NEISS and ICD-9-CM Injury Coding
Source: PLoS One. 2014 Mar 21;9(3):e92052. doi: 10.1371/journal.pone.0092052 (PMC3962381; doi:10.1371/journal.pone.0092052)
Supplement: Table S2 — Most common NEISS product codes among ICD-9-CM non-injuries. (DOCX) [file pone.0092052.s002.docx]

**Table S2. Most common NEISS product codes among ICD-9-CM non-injuries**

| **Product [Code]*** |
| --- |
| Diapers [1512] |
| Medical equipment, general [2400] |
| Liquid drugs (excluding aspirin, aspirin substitutes, iron preparations and antihistamines) [1927] |
| Other drugs or medications [1932] |
| Sports or recreation-related activity, excluding swimming [many product codes]** |
| Motor vehicles or parts (licensed; four or more wheels) [1901] |

*Product code definitions were obtained from the NEISS Product Comparability Table, 2012. Available: http://www.cpsc.gov//Global/Neiss_prod/comptable.pdf. Accessed 12 May 2012.

**NEISS product codes included in the sports or recreation-related activity category: 1211, 1205, 5401, 5034, 1282, 1276, 1266, 1267, 5030, 1261, 3235, 1206, 1212, 3254, 1270, 3265, 5017, 1264, 3283, 3247, 3255, 3216, 3297, 3217, 1279, 3245, 5032, 1295, 3272, 1235, 1215, 3222, 3256, 1269, 3284, 3236, 5016, 1237, 1392, 1338, 1200, 1677, 1645, 3299, 3277, 1272, 5040, 1233, 1333, 3289, 3290, 3291, 3274.
